# Supplementary material for: Yiai Fuzheng decoction inhibits triple-negative breast cancer by remodeling the immune microenvironment
Source: Front Immunol. 2025 Sep 30;16:1615631. doi: 10.3389/fimmu.2025.1615631 (PMC12518410; doi:10.3389/fimmu.2025.1615631)
Supplement: Supplementary file 2 [file DataSheet1.zip › Supplementary File 2/Treatment/C_vs_D/07_Pathway_Analysis/Pathway_Analysis/Sig_Pathways_ID/Glyoxylate and dicarboxylate metabolism.pdf]

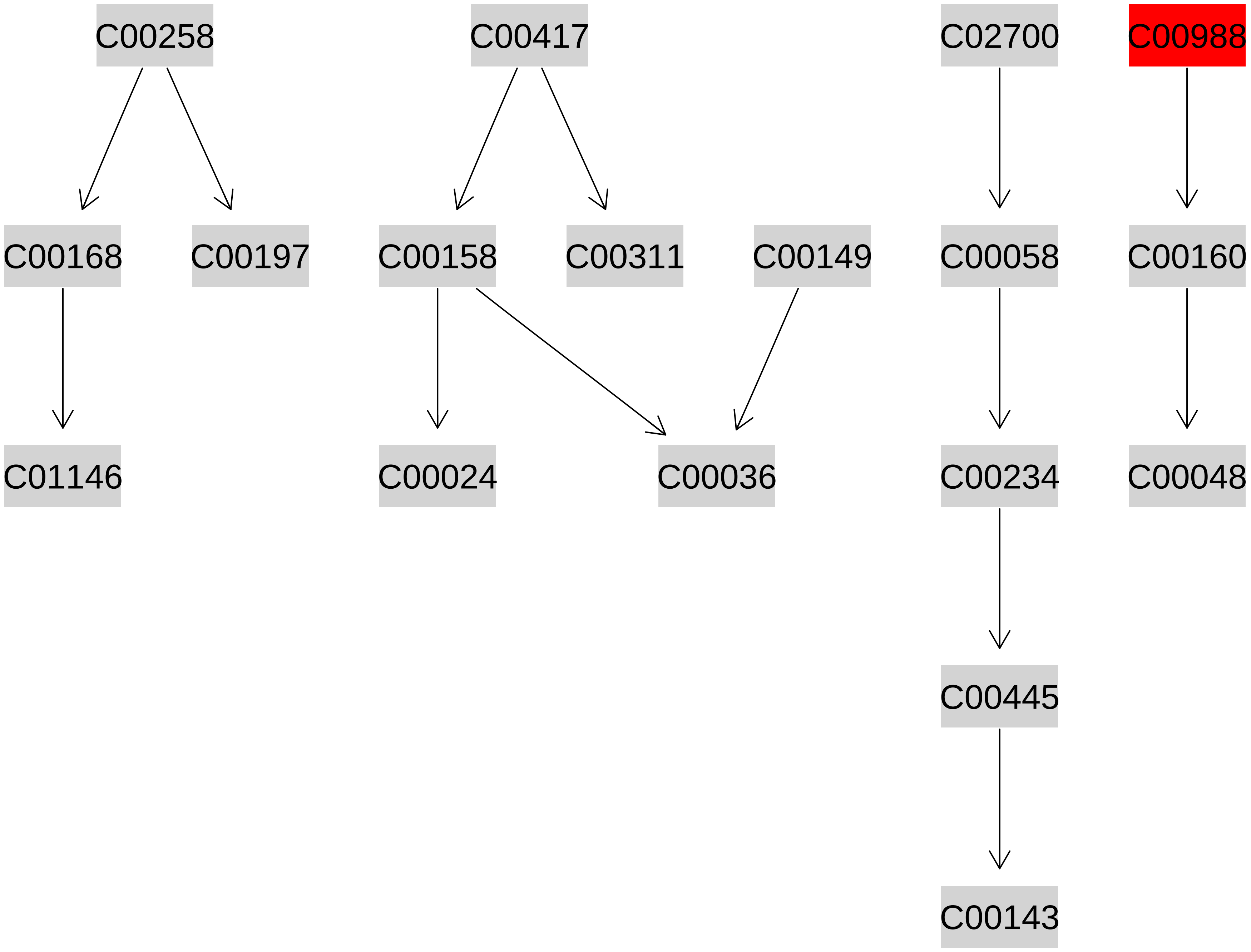
C00258
C00417
C02700
C00988
C00168
C00197
C00158
C00311
C00149
C00058
C00160
C01146
C00024
C00036
C00234
C00048
C00445
C00143
